# Symptoms of depression in ICU physicians

**DOI:** 10.1186/2110-5820-2-34

**Published:** 2012-07-27

**Authors:** Nathalie Embriaco, Sami Hraiech, Elie Azoulay, Karine Baumstarck-Barrau, Jean-Marie Forel, Nancy Kentish-Barnes, Frédéric Pochard, Anderson Loundou, Antoine Roch, Laurent Papazian

**Affiliations:** 1 Aix-Marseille University, URMITE CNRS-UMR 7278, 13005 Marseille, France; APHM, Hôpital Nord, Réanimation des détresses respiratoires et des infections sévères, 13015, Marseille, France; 2 Famirea Study Group, AP-HP, Hôpital Saint-Louis, Medical ICU, Université Paris-Diderot, Sorbonne Paris-Cité, Faculté de médecine, 1 avenue Claude Vellefaux, 75010, Paris, France; 3 Aix-Marseille University, Laboratoire de Santé Publique EA3279, 13005, Marseille, France; 4 APHM, Hôpitaux Sud, Service de Santé Publique et d’Information Médicale, 13009, Marseille, France; 5 Clinique du Chateau de Garches, Garches, France

**Keywords:** Intensive care unit, Organizational management, Conflict, Burnout, Depression, Physicians

## Abstract

**Background:**

Work and family are the two domains from which most adults develop satisfaction in life. They also are responsible for stressful experiences. There is a perception in the community that work is increasingly the source of much of our stress and distress. Depressive symptoms may be related to repeated stressful experiences. Intensive care unit (ICU) physicians are exposed to major stressors. However, the existence of depressive symptoms in these doctors has been poorly studied. This study was designed to evaluate the prevalence and associated risk factors of depressive symptoms in junior and senior ICU physicians.

**Method:**

A one-day national survey was conducted in adult intensive care units (ICU) in French public hospitals. Symptoms of depression were assessed using the Centers of Epidemiologic Studies Depression Scale (CES-D).

**Results:**

A total of 189 ICUs participated, and 901 surveys were returned (75.8% response rate). Symptoms of depression were found in 23.8% of the respondents using the CES-D scale. Fifty-eight percent of these intensivists presenting symptoms of depression wished to leave their job compared with only 33% of those who did not exhibit signs of depression as assessed by the CES-D scale (*p* < 0.0001). Multiple logistic regression showed that organizational factors were associated with the presence of depressive symptoms. Workload (long interval since the last nonworking weekend, absence of relief of service until the next working day after a night shift) and impaired relationships with other intensivists were independently associated with the presence of depressive symptoms. A high level of burnout also was related to the presence of depressive symptoms. In contrast, no demographic factors regarding ICU physicians and no factor related to the severity of illness of patients were retained by the model. The quality of relationships with other physicians (from other departments) was associated with the absence of depressive symptoms (protective effect).

**Conclusions:**

Approximately one of four intensivists presented symptoms of depression. The next step could be to test whether organization modification is associated with less depressive symptoms and less desire to leave the job.

## Background

Work and family are two domains from which most adults derive satisfaction in life; equally they are the common sources of stressful experiences. There is a perception in the community that work is increasingly the source of much of our stress and distress. Intensive care unit (ICU) physicians are generally perceived as very dedicated, perfectionist and exigent professionals. The culture of medicine accords low priority to physicians’ mental health.

Some studies have documented depressive and other psychiatric manifestations in women physicians and other professionals, and speculated that these symptoms are related to stress [1]. However, the prevalence of depressive symptoms in ICU practitioners has received little recent scientific attention. ICU doctors also are thought to be susceptible to “burnout,” a description for work-related distress that combines emotional exhaustion, depersonalization (treating people in an unfeeling, impersonal way), and a sense of low personal accomplishment [2]. Burnout is a problem that is specific to the work context, in contrast to depression, which tends to pervade every domain of a person’s life [3].

A recent report from the Centers for Disease Control and Prevention indicated that 9% of American adults met the criteria of current depression [4]. Coomber et al. [5] reported that approximately one third of U.K. ICU doctors appeared distressed and 10% reported depressive symptoms. Most of the attention has been focused on junior doctors and their long working hours. However, there also have been reports of distress among senior hospital doctors [6,7]. Because these factors are closely linked and because few studies have previously investigated depression in the intensive care environment, we designed a study to assess depressive symptoms among all intensivists (interns, residents, fellows, attending physicians). We hypothesized the following: 1) there is a high rate of depressive symptoms among intensivists; 2) presence of depressive symptoms in intensivists could be associated with the severity of illness of patients; and 3) depression in intensivists may be associated with organizational factors, such as workload and relationships with colleagues.

## Methods

This study related to the prevalence of depressive symptoms was an ancillary study of the survey dedicated to the burnout among French ICU physicians [2].

### Data collection

All directors of French adult ICUs from public hospitals were first contacted by letter and asked to participate in the study. In the agreement form, ICU directors had to indicate if they agreed to participate to the study and were asked to give the number of physicians (attending physicians, fellows, interns and residents) working in their ICU.

### Survey instrument

Each participating ICU received two types of documents validated by the members of the study board (NE, EA, KB, NKB, FP, AL, and LP). The first had to be completed by the director of the unit and was designed to describe the intensive care setting: information about the ICU; activity the year before (no. of admissions, duration of stay, Simplified Acute Physiology Score (SAPS) II score on admission, mortality); patients per nurse ratio; number of nurses and physicians who were on sick leave for more than 1 week the year before; presence of a discussion group and/or a psychologist in their unit.

The second document was a self-administered questionnaire for each physician working in the ICU. A covering letter outlining the purpose of the study along with a three-page questionnaire was sent to each participant. The letter also explained that the responses would be anonymous. The questionnaire was divided into four parts. Part 1 included basic demographic data, data concerning their professional activity, some questions about experiences during the past week (number of night shifts, number of their patients who died, number of decisions to withhold/withdraw treatment, conflict with other intensivists, ICU nurses, or patients’ families), and five questions about their situation the day of the survey: number of patients under his or her responsibility, night shift before the survey, on leave the day before the survey, probable death of a patient, decision of withholding/withdrawing treatment. Two more questions were asked about the number of conflicts with nurses and other intensivists the year before. Intensivists also were asked to rate their relationships with nurses, chief nurses, non-ICU physicians, and hospital management on a scale of 0 to 10. Intensivists were asked about their workload (mean number of work hours per week during the previous 6 months, mean number of night shifts per month the previous 6 months, time elapsed since their last week of holidays/last weekend off/last day off).

Part 2 consisted of the Maslach Burnout Inventory (MBI) scale. The MBI is a 22-item questionnaire that has been shown to be reproducible and valid [3,8]. The inventory asks respondents to indicate on a 7-point Likert scale (which does not include the word “burnout”) the frequency with which they experience certain feelings related to their work during the last week preceding the day of the survey. Burnout was defined as a high level of MBI. For the French population, a high level of burnout is defined by a MBI score higher than −8 [9].

Part 3 consisted of the Center for Epidemiologic Studies Depression Scale [10]. The Center for Epidemiologic Studies Depression Scale (CES-D) self-report includes 20 items comprising six subscales reflecting major dimensions of depression (depressed mood, feelings of guilt and worthlessness, feelings of helplessness and hopelessness, psychomotor retardation, loss of appetite, and sleep disturbances). Items refer to the frequency of symptoms during the last week and are scored on a 4-point scale ranging from 0 (rarely or none of the time) to 3 (most or all over the time). Question scores are summed to provide an overall score ranging from 0 to 60. Intensivists with scores of 19 or more for men and 23 or more for women were considered in the present study as presenting depressive symptoms. The CES-D was validated in French [11]. Due to the cross-sectional nature of this study, it is important to state that the single administration of the CES-D score does not represent a true diagnosis of depression, which is much more complex to establish. No services were provided for the respondents who presented depressive signs.

Part 4 of the survey consisted of seven questions regarding intensivists’ private lives using a 4-point categorical scale, as for the CES-D, from never to frequently. All respondents were asked to put their anonymous questionnaire in an envelope. In each center, all these envelopes were put in a single return envelope addressed to the researchers.

### Statistical analysis

Data are expressed as mean ± SD or median with interquartile range (IQR) according to the distribution of the data. One-way analysis of variance or Wilcoxon signed-rank test (according to the distribution of the data) was performed to compare continuous variables. To identify variables associated with depression, logistic regressions (forward-stepwise selection) were performed. All variables with a *p* value < 0.2 in the univariate analysis were entered in the model. The final models expressed the odds ratios (OR) and 95% confidence intervals (CI). Because some of the predictor variables used in the analyses was collected at the level of the ICU rather than the level of the physician, an analytic approach that incorporates a clustered design [generalized estimating equations (GEE) methodology)] was also used. A *p* value < 0.05 indicated significance. The statistical analyses were performed by using the SPSS software package version 15.0 (SPSS Inc., Chicago, IL).

## Results

A total of 189 of the 318 (59.4%) French ICU directors accepted to participate. A total of 1,189 surveys were sent on March 6, 2004, and the survey took place on March 25, 2004. A total of 901 surveys were returned (75.8% response rate).

### Characteristics

The characteristics of the ICUs and of the respondents are developed in Tables 1 and 2. Respondents were mainly attending physicians (62%). Their mean age was 39 ± 10 years. Approximately one-half of the intensivists (52%) worked in teaching hospitals. They declared to work 59 ± 12 hours per week, to do 4.8 ± 2.0 night shifts per month, and to be working in the ICU for 60 (range, 10–180) months.

**Table 1:** Characteristics of the 189 participating ICUs

Annual number of admissions to the ICU*	600 ± 310
Mean duration of hospitalization in the ICU, days*	7.9 ± 2.6
SAPS II score on admission*	39 ± 5
ICU mortality, %*	19.3 ± 5.5
Staffing, patients per nurse, n	3.0 ± 0.6
No. of physicians in charge of the ICU	3.6 ± 2.2
No. of interns or residents per ICU	2.7 ± 2.0
No. of ICU beds the day of the survey	14 ± 7

*Year 2003. Values are expressed as mean (±SD).

**Table 2:** Characteristics of participants

N	901
Age, yr (mean ± SD)	39 ± 10
Women, %	28
Married or with a partner, %	70
Number of children (mean ± SD)	1.2 ± 1
At least one child, %	58
Practicing a religion, %	34
Traveling time to work, min [median (IQR)]	17.5 (10–30)
Status	
· Interns/residents, %	24
· Fellows, %	14
· Attending physicians, %	62
Teaching hospital, %	52
Working hours per week, hours (mean ± SD)	59 ± 12
ICU practice, months [median (IQR)]	60 (10 – 180)
High level of burnout, %	46
Full-time ICU activity, %	70
Night shifts per month, number	4.8 ± 2.0
Compensation for overtime, %	33
Relief of service until the next working day after a night shift %	45
**During the last 7 days:**	
· Death of one of your patients, %	68
· Withholding decided on your own, %	10
· Withholding decided by the team, %	67
· Night shift, %	
o 0	16
o 1	36
o 2	36
o ≥3	13
**The day of the survey:**	
· Night shift before the survey, %	16
· off the day before, %	18
· Withholding/withdrawing, %	27
· Probable death of one of your patients, %	46
Period since the last non-working day, days [median (IQR)]	4 (3–6)
Period since the last non-working week, days [median (IQR)].	40 (20–90).

### Prevalence of the symptoms of depression in intensivists and desire to leave the job

Mean CES-D scale score was 14.2 ± 9.2 for the entire population. Finally, 23.8% of the respondents were included in the group of intensivists presenting depressive symptoms. As expected, mean CES-D scale score was higher for intensivists included in the “presence of depressive symptoms” group than in the absence of depressive symptoms (27.5 ± 6.9 vs. 10 ± 4.9, respectively; *p* < 0.0001).

Fifty-eight percent of these intensivists presenting symptoms of depression wished to leave their job compared with only 33% of those who did not exhibit signs of depression as assessed by the CES-D scale (*p* < 0.0001).

**Figure 1:**
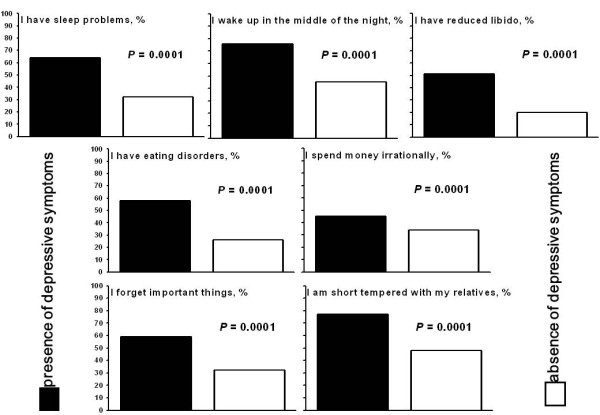
Impact of depressive symptoms on daily life.

As shown in Figure 1, the respondents who presented symptoms of depression reported more sleep problems, more eating disorders and more altered relationships with their relatives than intensivists who did not present such symptoms.

### Factors associated with the presence of depressive symptoms

#### Univariate analysis (Tables 3 and 4)

**Table 3:** Presence of depressive symptoms according to demographic and factors associated with the severity of illness of patients

	**Presence of depressive symptoms N = 214**	**Absence of depressive symptoms N = 687**	***P***
Age, yr (mean ± SD)	41 ± 10	39 ± 10	0.14
Women, %	23	29	0.11
Married or with a partner, %	70	71	0.89
Number of children, %			
· 0	39	43	0.32
· ≥1	61	57	
Practicing a religion, %	37	33	0.32
Travelling time to work, min (mean ± SD)	24 ± 17	23 ± 18	0.37
Status			
· Interns/residents or fellows, %	34	40	0.14
· Attending physicians, %	66	60	
ICU practice, mo [median (IQR)]	84 (18–190)	55 (6–160)	0.03
ICU mortality, year 2003, %	18.5 ± 5.5	19.6 ± 5.8	0.02
SAPS II score, year 2003	39 ± 5	40 ± 5	0.16
**During the last 7 days:**			
· Death of at least one of your patients, %	69	67	0.58
· Withholding decided of your own, %	13	10	0.23
· Withholding decided by the team, %	65	67	0.51
**The day of the survey:**			
· Withholding/withdrawing, %	30	27	0.29
· Probable death of one of your patients,%	48	46	0.55

Data are mean ± SD or median (IQR) unless otherwise indicated.

**Table 4:** Presence of depressive symptoms and organizational factors

	**Presence of depressive symptoms N = 214**	**Absence of depressive symptoms N = 687**	***P***
**Unit factors**			
Teaching hospital, %	53	47	0.17
Number of ICU beds*	14.3 ± 7.3	15.7 ± 9.1	0.04
Patients admitted, year 2003, number*	579 ± 306	619 ± 318	0.13
Intensivists (excluding interns and residents) working in the ICU, number	6.0 ± 2.4	6.0 ± 2.6	0.95
Intensivist absenteeism for more than 1 week, year 2003, %	39	32	0.09
Compensation for overtime, %	30	34	0.23
Relief of service until the next working day after a night shift, %	36	48	0.002
Discussion group, %	39	43	0.29
Psychologist, %	23	25	0.62
**Intensivist factors**			
Working hours per week, hours*	60 ± 13	58 ± 12	0.03
Full-time ICU activity, %	72	69	0.47
High level of burnout, %	81	36	0.0001
Night shifts per month, number*	5.1 ± 1.9	4.7 ± 1.9	0.008
Period since the last nonworking day, days**	4 (3–8)	4 (3–6)	0.002
Period since the last nonworking weekend, days**	10 (4–19)	7 (4–15)	0.0001
**During year 2003:**			
· No. of conflicts with a nurse**	2 (0–5)	2 (0–4)	0.23
· No. of conflicts with a colleague intensivist**	2 (1–5)	1 (0–3)	0.0001
**During the last 7 days:**			
· Night shift, %			0.07
- 0	12	17	
- 1	32	37	
- 2	42	34	
- ≥3	14	12	0.001
· Conflict with a nurse, %	19	11	0.0001
· Conflict with a colleague intensivist, %	29	12	0.0001
· Conflict with a patient’s family, %	11	7	0.07
**The day of the survey:**			
· Night shift before the survey, %	19	15	0.16
· Off the day before, %	14	19	0.10
· Patients-to-intensivist ratio*	7.3 ± 4.7	7.5 ± 4.9	0.56

Whereas age and status were not associated with depressive symptoms, respondents with depressive symptoms had a longer ICU practice than those not presenting such symptoms. No factor reflecting the severity of illness as well as patients’ deaths and decision of withholding/withdrawing had an impact on the presence of depressive symptoms. The presence of a discussion group and/or a psychologist was not associated with less depressive symptoms. In contrast, there were more intensivists with a high degree of burnout in the group presenting depressive symptoms. To be relieved of service until the next working day after a night shift was protective (decreased rate of depressive symptoms). The workload was associated with the presence of depressive symptoms (working hours per week, number of night shifts per month, delay from the last working day, weekend, or week).

**Figure 2:**
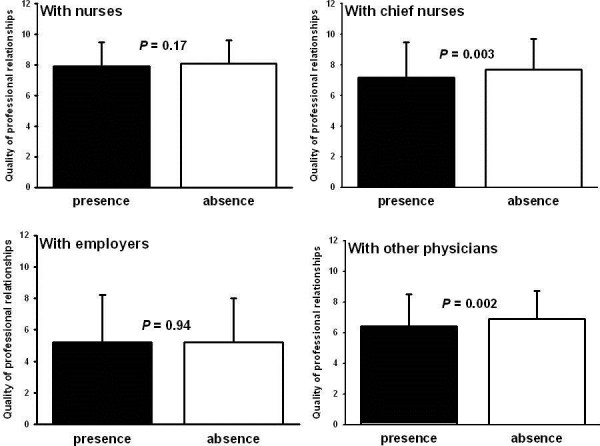
**Relationships with other professionals.** Professional relationships are quote on a scale of 0 to 10. Values are expressed as mean (SD).

Intensivists with depressive symptoms declared more conflicts with nurses or colleagues during the previous 7 days. Finally, relationships with other nonintensivists colleagues and chief nurses were worse for ICU physicians experiencing depressive symptoms (Figure 2).

#### Multivariate analyses

Multivariate analyses were performed to evaluate the independent relationship between the presence of depressive symptoms and all factors with a *p* value < 0.2 by univariate analysis. Multiple logistic regression (Table 5) showed that the presence of a high level of burnout was strongly related to the presence of depressive symptoms. In contrast, no demographic factors regarding ICU physicians and no factor related to the severity of illness of patients were retained by the model. Organizational factors were in contrast associated with the presence of depressive symptoms. Workload (a long period from the last nonworking weekend, absence of relief of service until the next working day after a night shift) and impaired relationships with other intensivists were independently associated with the presence of depressive symptoms. In contrast, the quality of the relationships with other physicians (from other departments) was associated with the absence of depressive symptoms (protective effect). Multivariate analysis using a GEE methodology identified the same independent factors associated with the presence of depressive symptoms (Table 5).

**Table 5:** Multivariate analyses: factors associated with the presence of depressive symptoms

	**Logistic regression**		**GEE**	
**Variable**	**OR (and 95% CI)**	***P *value**	**OR (and 95% CI)**	***P *value**
**Demographic factor:**				
None				
**Intensivist factor:**				
- High level of burnout*	6.79 (4.13-11.18)	0.0001	6.82 (4.5-10.33)	0.0001
**Organizational factors:**				
- Relief of service until the next working day after a night shift*	0.61 (0.38-0.99)	0.047	0.64 (0.41-0.99)	0.04
- Period since the last nonworking weekend, for each day	1.023 (1.004-1.041)	0.016	1.023 (1.006-1.04)	0.009
- Conflict with a colleague intensivist during the last 7 days*	2.52 (1.49-4.26)	0.001	2.47 (1.41-4.34)	0.001
- Relationships with other physicians, for each additional point of the 0–10 rating scale	0.89 (0.79-0.99)	0.043	0.88 (0.78-0.99)	0.049

*,Dichotomized variable.

**Continuous variables included in the model:** age; working hours per week; ICU practice; night shifts per month; period since the last nonworking weekend; relationships with nurses; relationships with chief nurses; relationships with other physicians; number of ICU beds; patients admitted, year 2003; ICU mortality, year 2003; SAPS II score, year 2003.

**Categorical variables included in the model:** sex; status (interns/residents or fellows-attending physicians); intensivist absenteeism for more than one week, year 2003; conflict with a nurse during the last 7 days; conflict with a colleague intensivist during the last 7 days; conflict with a patient’s family during the last 7 days; teaching hospital; relief of service until the next working day after a night shift; off the day before the survey; night shift before the survey; high level of burnout.

## Discussion

As expected, this study reported a high rate of depressive symptoms among intensivists. The presence of depressive symptoms was not associated with patients’ severity of illness. In contrast, depression in intensivists was associated with organizational factors, such as workload and relationships with colleagues.

Approximately a quarter of the physicians included in the present study exhibited signs of depression. Coomber et al. [5] reported that 12% of U.K. ICU physicians showed clinically important levels of depression. In a 2002 survey of internal medicine trainees, 40% of female residents and 32% of male residents reported four or five symptoms of depression [12]. It is very interesting to observe that the respondents with depressive symptoms included in the present study had a longer ICU practice compared with those who had not.

In the general population, risk factors for major depression include being a woman [13]. Frank and Dingle reported that women physicians with histories of self-identified depression shared many attributes, such as not being partnered, or being dissatisfied with career and work [14]. This was not the case in the present study where gender was not associated with depressive symptoms.

In the study by Coomber et al. [5], there was no relationship between the level of depression and the age, the reported hours worked in the previous week, or the number of ICU beds for which they were responsible. This lack of association with work hours has been noted so far [15]. In the present study, we did not report any association between work hours and depression. However, there was an inverse relationship between symptoms of depression and the delay since the last nonworking weekend, suggesting that a sufficient period of rest is probably a more important factor than the number of work hours. ICU organizational aspects therefore are very important and could be a major issue in decreasing physicians’ depression. Chronic sleep deprivation may be a contributive factor that explains such a high rate of physicians presenting depressive symptoms [16-18]. Longer resting periods could be therefore useful to improve physicians’ psychological health. Depressive disorders are likely associated to job-related burnout. More specifically some studies suggest that burnout leads to depressive symptoms [19,20]. In the present study, the presence of a high level of burnout was strongly related to the presence of depressive symptoms.

The limitations of this study include the biases of self-reporting (e.g., skipping questions, nondisclosure, same day of completion of the questionnaire by the intensivists and different interpretations of meaning), the lack of standardized interviews or specific criteria for making psychiatric diagnoses, incomplete information (e.g., no items on mental health treatment). The responders may not represent the whole French ICU physician community in terms of psychiatric morbidity, but we had no way of exploring the state of mental health of the nonresponders. Nonetheless, we would argue that the level of morbidity we have detected is likely to be a conservative estimate, because it can be argued that nonresponse is associated with “burnout” and “depression.” However, we do recognize that such conjecture needs further investigation, because nonresponse may be simple disinterest rather than overwork and lack of time. The study was done in 2004. The working conditions may have change since 2004, in particular concerning the number of ICU physicians who leave the hospital after a night shift. This could have modified the prevalence of depressive symptoms. However, we have no available information to indicate that the rate of intensivists leaving the hospital early in the morning following a night shift has increased recently. Additional investigations are necessary to design appropriate interventions that could be implemented to decrease depression rate in ICU doctors.

It is important to diagnose and to treat depression, because treatment is associated with improved work productivity [21], and with a reduction of suicide [22]. Interventional studies designed to evaluate whether alteration of ICU organization is able to decrease the prevalence/incidence of depressive symptoms are warranted. It is an important objective individually (to increase the intensivist well-being) but also collectively (ICU caregivers and patients) by potentially reducing intensivists’ turnover.

## Abbreviations

ICU, Intensive care unit; CES-D, Centers of Epidemiologic Studies Depression Scale; SAPS II, Simplified Acute Physiology Score; MBI, Maslach Burnout Inventory; IQR, Interquartile range; GEE, Generalized estimating equations.

## Competing interests

The authors declare that they have no competing interests.

## Authors’ contributions

Study concept and design: Embriaco, Azoulay, Pochard, Kentish-Barnes, Papazian, Acquisition of the data: Embriaco. Analysis and Interpretation of the data: Embriaco, Loundou, Barrau, Azoulay, Papazian. Drafting of the manuscript: Embriaco, Hraiech, Papazian. Critical revision of the manuscript: Forel, Roch. Dr. Papazian, Dr Embriaco and Mr. Loundou had full access to all the data in the study and take responsibility for the integrity and accuracy of the data. This manuscript was edited for proper English language, grammar, punctuation, spelling, and overall style by one or more of the highly qualified native English-speaking editors at American Journal Experts. All authors read and approved the final manuscript.
